# Magnetic bentonite decorated with Pd nanoparticles and cross-linked polyvinyl pyridine as an efficient nanocatalyst for Suzuki coupling and 4-Nitrophenol reduction reactions

**DOI:** 10.1038/s41598-023-27800-3

**Published:** 2023-02-03

**Authors:** Haniyeh Daneshafruz, Pourya Mohammadi, Hossein Barani, Hassan Sheibani

**Affiliations:** 1 Department of Chemistry, Shahid Bahonar University of Kerman, Kerman, 76169 Iran; 2 Department of Carpet, University of Birjand, 17 Shahrivar Street, Birjand, Iran

**Keywords:** Chemistry, Catalysis, Organic chemistry

## Abstract

This study reports the preparation of a novel type of support based on magnetically recyclable bentonite functionalized with divinylbenzene-polyvinyl pyridine (PVP-DVB) for Pd (II) nanocatalyst by a simple cost-effective method. Firstly, the conventional co-precipitation method synthesized Fe_3_O_4_ nanoparticles (NPs) onto bentonite sheets. Then the prepared magnetic support surface was functionalized by divinylbenzene-polyvinyl pyridine (PVP-DVB) to create a cross-linked polymer with a high coordination ability with palladium. Repeated nitrogen units in the PVP-DVB polymer chain increase the number of Pd bonds and thus lead to higher performance of the nanocatalyst. Finally, the palladium NPs were simultaneously synthesized and immobilized under mild conditions. The synthesized nanocatalyst was characterized by several methods such as scanning electron microscopy, transmission electron microscopy, X-ray photoelectron spectroscopy, X-ray diffraction, Fourier-transform infrared spectroscopy, vibrating sample magnetometer, inductively coupled plasma mass spectrometry and thermogravimetric analysis. The efficiency of synthesized heterogeneous nanocatalyst was investigated in Suzuki–Miyaura cross-coupling reactions between a range of aryl halides (X = Cl, Br, I) with phenylboronic acid and in the reduction of 4-nitrophenol (4-NP). Moreover, the synthesized nanocatalyst could be easily recovered and reused several times with an efficiency greater than 90%.

## Introduction

Today, the Suzuki-Miyaura coupling reaction is used as a modern organic method in the presence of metal nanoparticles such as Pd for the synthesis of biaryl compounds, which are used in the manufacture of many drugs, polymers, and natural products^1,2^. One of the main reasons for the popularity of Suzuki reactions is the non-toxicity of the used materials and environmentally friendly solvents^3,4^. Accordingly, in recent years, the design and synthesis of homogeneous or heterogeneous catalysts with unique features such as recyclability and compatibility with the environment with maximum efficiency in increasing the speed of this reaction have been one of the main concerns of the industry^5,6^. Palladium is known as an expensive metal and the main catalyst in coupling reactions. Pd nanoparticles have many advantages, which easy access to d-layer electrons, distinct quantum properties, and tunable size are the most prominent but it is not used as a homogeneous catalyst because a significant amount of it is wasted during the separation process, therefore, to solve this problem, it is used as a heterogeneous metal catalyst on some supporting compounds^7–9^. It is very important to choose cheap, available, and environmentally friendly supports from the point of view of green chemistry. Also, the support plays a key role in the preparation of heterogeneous catalysts because the poor performance of the catalyst or its lack of recovery can be a consequence of the weak interaction of the support with metal ions^10^. Bentonite is a type of clay and a natural and non-toxic mineral material that, with an optimal surface and structure, can be a suitable support and at the same time an effective adsorbent to maintain polymer and transition metals on its surface.

On the other hand, the indiscriminate release of nitroaromatics in water as emerging pollutants that are widely used by industrialists is considered a serious threat to the environment and human health. 4-NP as a nitroaromatic causes headache, nausea, drowsiness, and cyanosis in humans^11,12^. Hence, a wide range of methods such as surface adsorption, membrane separation, electrocoagulation, and biological treatment has been developed to remove 4-NP from water but catalytic reduction can be named as the best-known method because it is both economical and very safe. The amines obtained during this reduction are valuable raw materials or intermediates in the production of drugs, rubber, dyes, and antioxidants^13,14^.

Therefore, considering the importance of catalysts and following the previous works^15–17^, in this study, after preparing an efficient substrate for the stabilization of palladium nanoparticles using non-toxic compounds such as bentonite clay and PVP-DVB with an active surface and high thermal stability, used for Suzuki- Miyaura reactions between a range of aryl halides with phenylboronic acid and reduction of 4-NP in the presence of NaBH_4_. This magnetic catalyst can be easily separated from the reaction solution using a magnet several times without reducing its catalytic performance.

## Experimental

### Material and methods

Bentonite clay, PVP-DVB, palladium chloride (PdCl_2_), FeCl_2_.4H_2_O, FeCl_3_.6H_2_O, ethanol (EtOH), acetic acid, hydrazine hydrate (NH_2_NH_2_), ammonia, phenylboronic acid, iodobenzene, chlorobenzene, bromobenzene, 4-nitrophenol (4-NP), sodium borohydride (NaBH_4_), potassium carbonate (K_2_CO_3_), and acetonitrile (MeCN), were obtained from Merck and Sigma-Aldrich. The prepared nanocatalyst was characterized by scanning electron microscopy (FE-SEM, TESCAN-MIRA3), transmission electron microscope (TEM, EM10 c–100 kV), and Fourier-transform infrared spectroscopy (FT‐IR, Bruker, Germany, RT-DLATGS detector). Nanocatalyst surface images and EDX-MAP spectra were obtained using the TESCAN MIRA III. Thermogravimetric analysis (TGA) was carried out by a thermal analyzer with a 20 °C/min heating rate in the temperature range of 25 to 1000 °C under compressed nitrogen flow. Also, the chemical composition of the nanocatalyst surface was analyzed using an X-ray photoelectron spectrometer (XPS, SPECS model UHV analysis system). The magnetic property of the prepared nanocatalyst was measured by a vibrating sample magnetometer (VSM) and the percentage of palladium metal immobilized on the substrate was measured using ICP analysis with ARL Model 3410. Finally, the reduction of 4-NP in the presence of the synthesized nanocatalyst was controlled by UV–vis spectroscopy.

### Synthesis of Fe_3_O_4_/bentonite (Fe-Ben)

At first, 0.5 g of bentonite clay was dispersed in H_2_O solvent (120 mL) for 30 min by ultrasonic, and then 2.7 g of FeCl_3_.6H_2_O was added to the above solution. After one minute 1.0 g of FeCl_2_.4H_2_O was added to the solution and stirred for 3 h. After this time, 11 mL of 25% NH_3_ solution was injected into the solution in about 11 seconds at 60 °C, and then it was stirred for another hour. The resulting magnetic compound was isolated by a magnet and washed 4 times with H_2_O and finally, the product was dried at 25 °C for overnight.

### Synthesis of Fe-Ben /PVP-DVB composites

First, 2.0 g of PVP-DVB was dispersed in 50 mL of ethanol solvent along with stirring and then 1 mL of CH_3_COOH was added to the mixture. This mixture was stirred for 5 h at 60 °C. The resulting precipitate was separated by filtration and washed 3 times with EtOH. After drying the precipitate at room temperature, 0.75 g of it was mixed in 50 mL of DMSO solvent. While 1.0 g of Fe-Ben was stirred separately in DMSO for 20 min. Finally, two solutions were added to each other and stirred for 24 h at 60 °C. The resulting magnetic composite was removed by an external magnet, rinsed twice with ethanol, and dried at room temperature.

### Synthesis of Fe-Ben/PVP-DVB/Pd nanocatalyst

**Figure 1:**
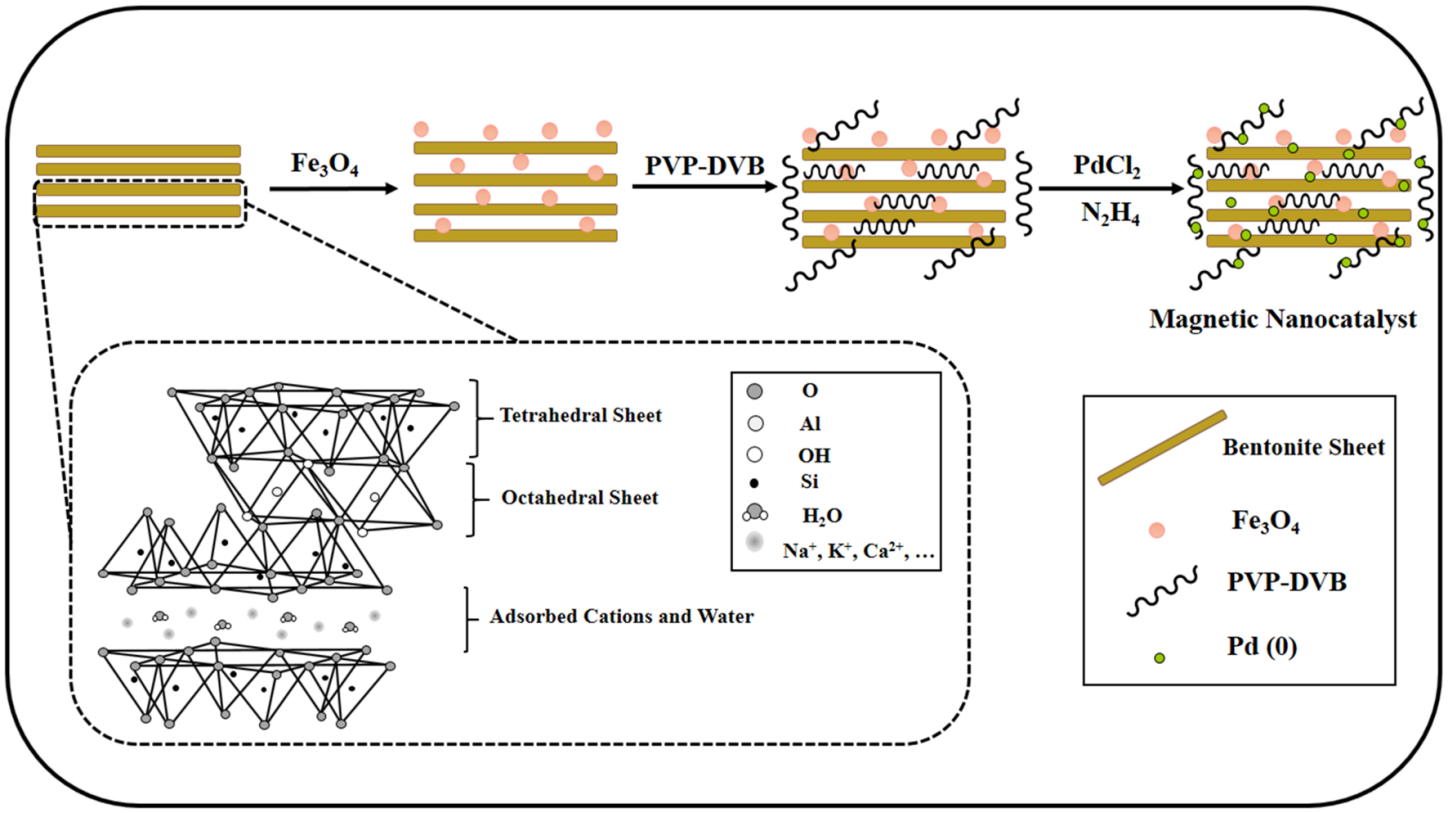
Schematic of preparation of Fe-Ben/PVP-DVB/Pd nanocatalyst.

Typically, 0.03 g of palladium (II) chloride was stirred in 70 mL of acetonitrile for 2 h until all palladium particles were dissolved, and then a clear yellow solution was obtained. Also, 0.50 g of Fe-Ben /PVP-DVB composite (which was prepared in the previous step) was stirred in 50 mL of acetonitrile for 30 min. These two prepared mixtures were added together and stirred at 60 °C for 24 h. After that, 1 mL of hydrazine hydrate solution (0.5 mL hydrazine hydrate (80%) in 5 mL of ethanol) was injected into the above reaction mixture. After 6 hours, the synthesized nanocatalyst was collected by a magnet and washed twice times with ethanol. Finally, the nanocatalyst was dried at 25 °C (Fig. 1).

### Procedure of Suzuki–Miyaura reactions

**Figure 2:**
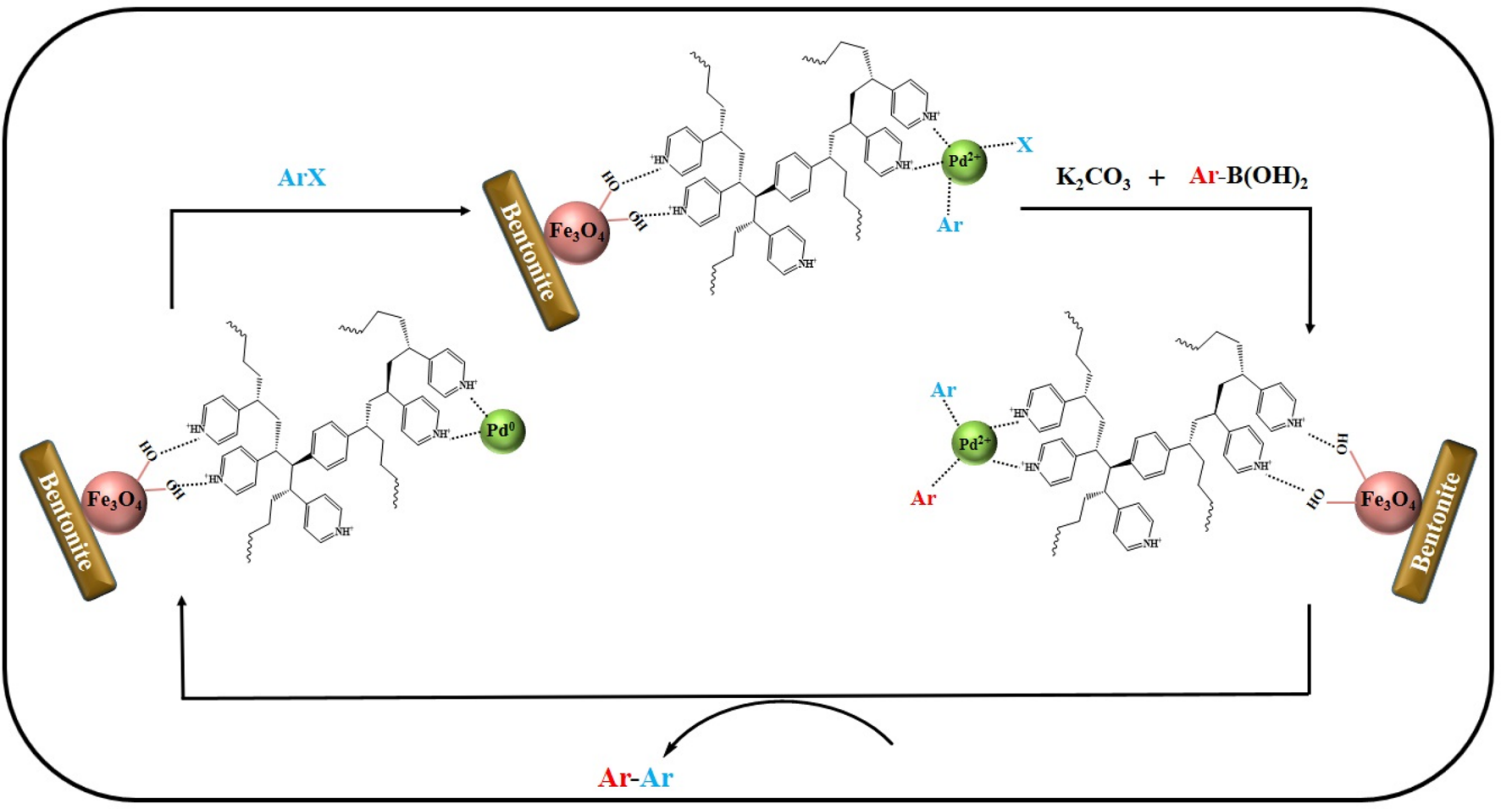
A possible mechanism of Suzuki coupling reaction catalyzed by Fe-Ben /PVP-DVB/Pd nanocatalyst.

To perform the Suzuki coupling reaction (Fig. 2), 0.5 mmol of aryl halide was first dissolved in 2 mL of EtOH/H_2_O solvent mixture (v/v = 1:1) in a 25 mL round-bottom flask and immediately 0.073 g of phenylboronic acid (0.6 mmol) with 0.06 g of potassium carbonate (2.0 mmol) and 10 mg (0.3 mol% Pd) of nanocatalyst was added to this flask. This mixture was stirred at high speed at 60 °C until the completion of the reaction (detection by TLC) and then the reaction mixture temperature was decreased to ambient temperature, the nanocatalyst was recycled using a magnet. On the other hand, the obtained products were extracted using dichloromethane (three times, 10 mL). The solvent evaporated and the products were dried over dry sodium sulfate. In the end, the final product was purified using column chromatography.

### General procedure for 4-NP reduction

**Figure 3:**
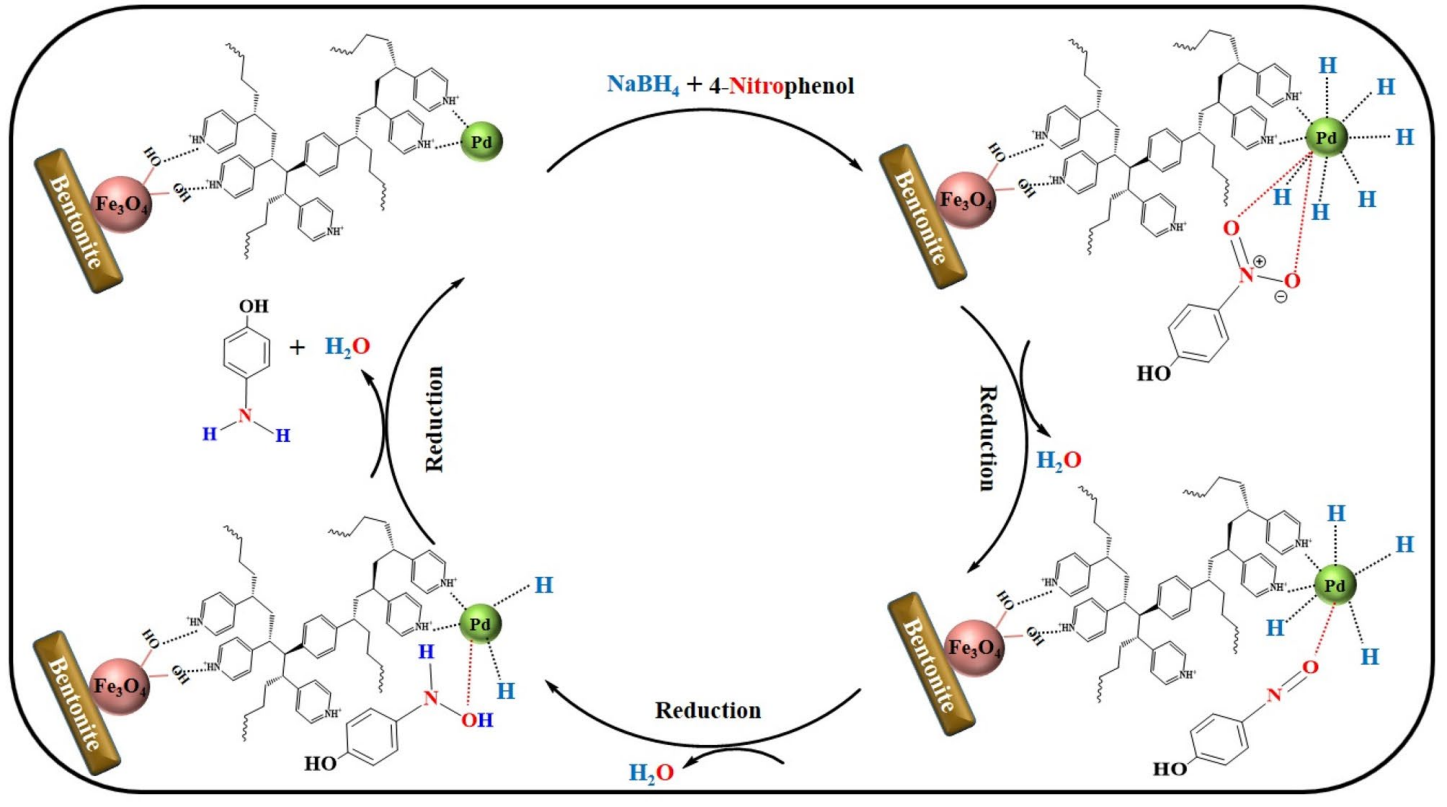
A possible mechanism for the catalytic reduction of nitroarene compounds by recyclable Fe-Ben /PVP-DVB/Pd nanocatalyst.

To perform the reduction of 4-NP by the synthesized nanocatalyst (Fig. 3), first, 10 mL of 2.5 mM solution of 4-NP and 10 mL of 250 mM NaBH_4_ solution were freshly prepared. Then 4 mL of water, 0.5 mL of NaBH_4_, and 0.1 mL of 4-NP were poured into a quartz cuvette, respectively. To start the reaction, 2 mg (0.06 mol% Pd) of the synthesized nanocatalyst was first added to the cuvettes and the mixture was stirred vigorously at room temperature. A UV–vis spectrophotometer was used to monitor the reduction of 4-NP to 4-AP, and the absorption intensity of 4-NP was recorded at a maximum wavelength of 400 nm. At the end of the work, the magnetic nanocatalyst was reused from the reaction medium by an external magnetic field and washed for reuse. All these steps were repeated for 1 mg (0.03 mol% Pd) of nanocatalyst.

### Ethical approval

This article does not contain any studies with human participants or animals performed by any of the authors.

### Consent to participate

We confirm that the manuscript has been read and approved by all named authors and that there are no other persons who satisfied the criteria for authorship but are not listed. We further confirm that the order of authors listed in the manuscript has been approved by all of us.

## Results and discussion

### Characterization studies of Fe-Ben/PVP-DVB/Pd nanocatalyst

We designed the Fe_3_O_4_ and Pd nanoparticles immobilized on bentonite-PVP cross-linked composite (Fe-Ben/PVP-DVB/Pd nanocatalyst) that the nanoparticles were synthesized by using the coprecipitation and reduction methods, respectively. The modification of the layers of bentonite by PVP cross-linked was done in order to the stronger connections between them, and also caused the increase of centers of acceptors to increase of loading Fe_3_O_4_ and Pd nanoparticles. Finally, a prepared catalyst as an efficient nanocatalyst was applied for the Suzuki coupling and reduction of 4-NP reactions.

### FT-IR

**Figure 4:**
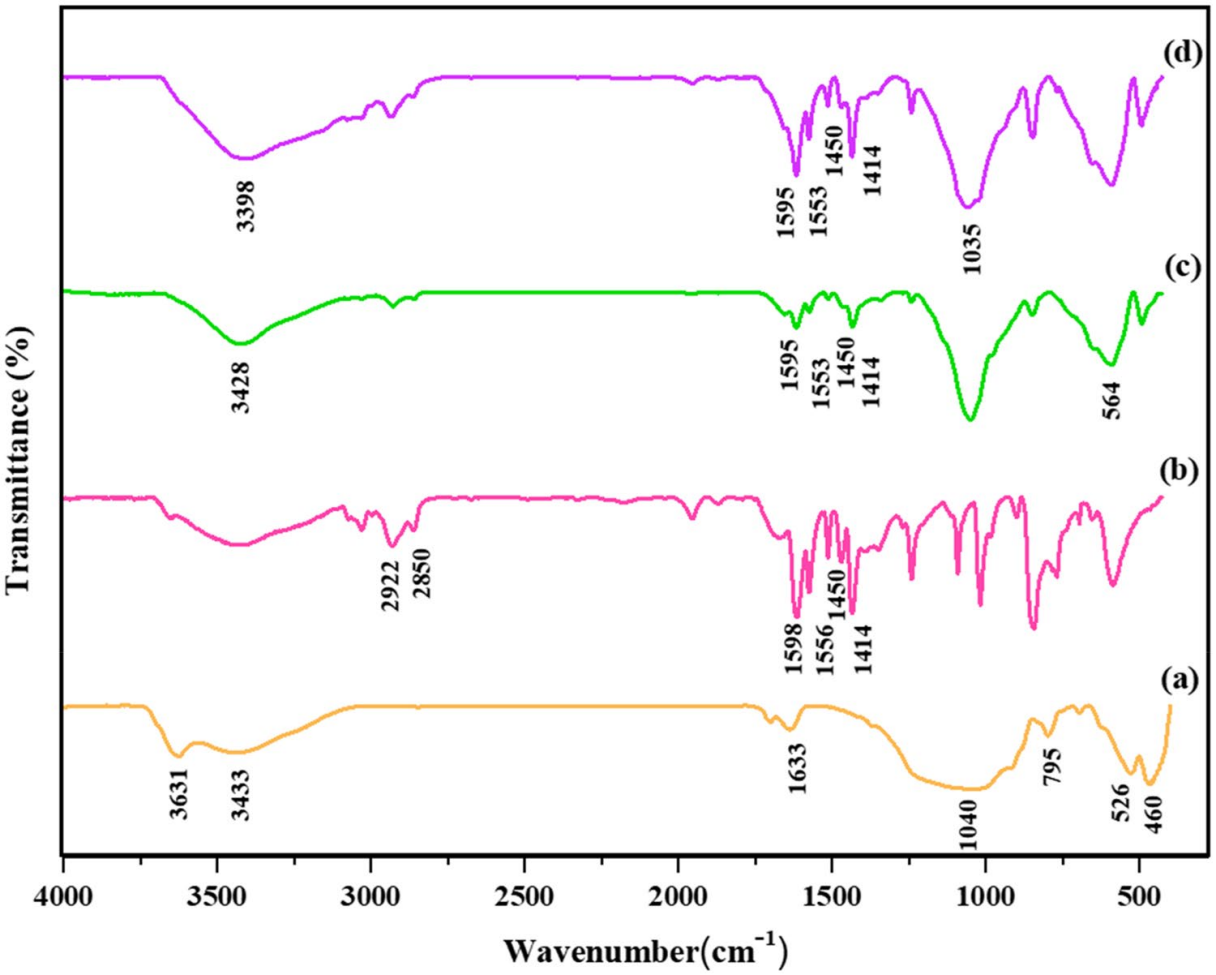
Normalized FT-IR spectra of (**a**) bentonite, (**b**) PVP-DVB, (**c**) Fe-Ben/ PVP-DVB_,_ and (**d**) Fe-Ben/PVP-DVB/Pd nanocatalyst.

FT-IR analysis was used to identify and evaluate the functional groups of the prepared nanocatalyst. In this regard, spectra comparison of (a) bentonite, (b) PVP-DVB, (c) Fe-Ben/PVP-DVB, and (d) Fe-Ben/PVP-DVB/Pd was shown in Fig. 4. The pristine bentonite spectrum showed specific peaks of about 460 cm^−1^, 795 cm^−1^, and 1040 cm^−1^, indicating stretching vibrations (Si–O–Si) in the structure of bentonite. The absorption band at 1633 cm^−1^ could also be attributed to the bending vibrations of hydroxyl groups (O–H) in bentonite. The absorption peak at 526 cm^−1^ corresponds to the stretching vibration of Al–O–Si–O which indicated the presence of feldspars. The presence of adsorbed water was revealed by the peaks that appeared at 3433 cm^−1^ and 3631 cm^−1^ ^18,19^. The IR spectrum of PVP-DVB shows the vibrations of the pyridine ring were dedicated by absorption bonds at 1414 cm^−1^, 1450 cm^−1^, 1556 cm^−1^, and 1598 cm^−1^. There were also two prominent peaks with the centrality of 2850 cm^−1^ and 2922 cm^−1^ that could be attributed to the stretching vibrations of –CH_2_– in the skeleton of the polymer. After increasing PVP-DVB and Fe_3_O_4_ on the surface of bentonite (Fig. 4c), specific peaks related to the stretching vibrations of the pyridine ring appeared in the region 1414 cm^−1^ and1598 cm^−1^ and also peak at 564 cm^−1^ related to the stretching vibration of Fe–O magnetic nanoparticles which confirmed the effective presence of these compounds on the surface of bentonite^20^. The IR spectrum of the synthesized nanocatalyst was shown in Fig. 4d. The interaction of functional groups with palladium metal has caused the bonds to become more polarized and as a result, the intensity of the peaks has been strengthened^21,22^.

### Electron microscopy and elemental analysis

**Figure 5:**
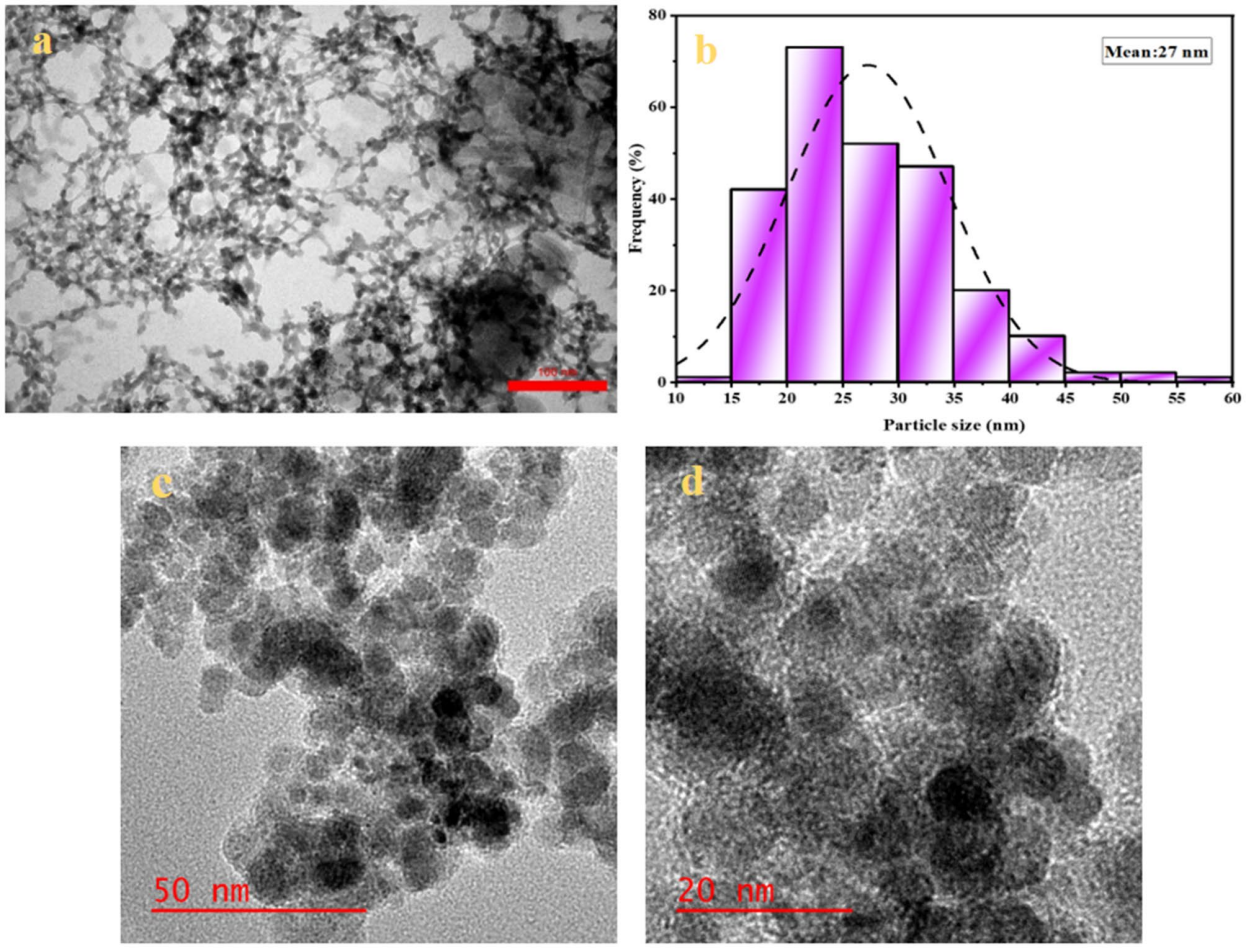
TEM (**a**), HRTEM (**c** & **d**), and particle size distribution histogram (**b**) of synthesized Fe-Ben/PVP-DVB/Pd nanocatalyst.

The morphology and structure of Fe-Ben/PVP-DVB/Pd nanocatalyst were determined by TEM (Fig. 5a) and HRTEM (Fig. 5c and 5d). Morphological analysis showed that Pd nanoparticles are quasi-spherical and uniform and without any cumulatively distributed. In addition, the histogram of nanoparticles size distribution determined from TEM images is shown in Fig. 5b. According to this histogram, the average particle diameter was determined to be about 27 nm.

**Figure 6:**
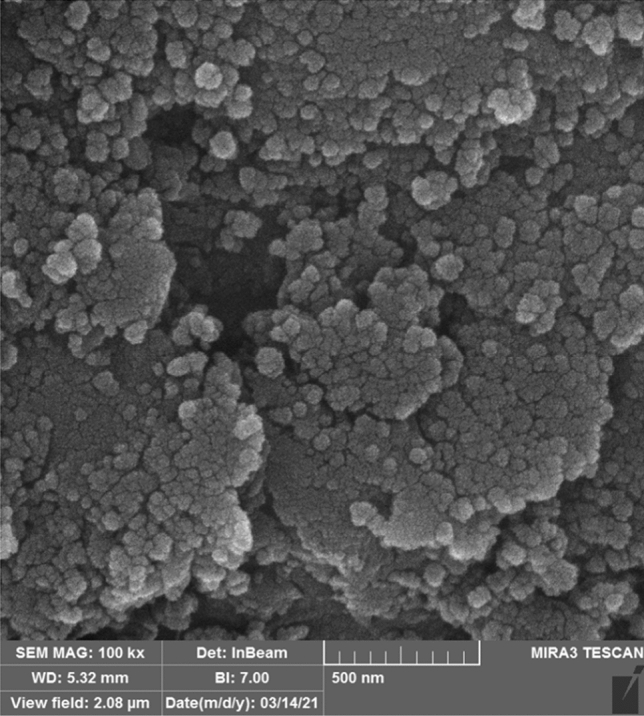
SEM image of Fe-Ben/PVP-DVB/Pd nanocatalyst.

**Figure 7:**
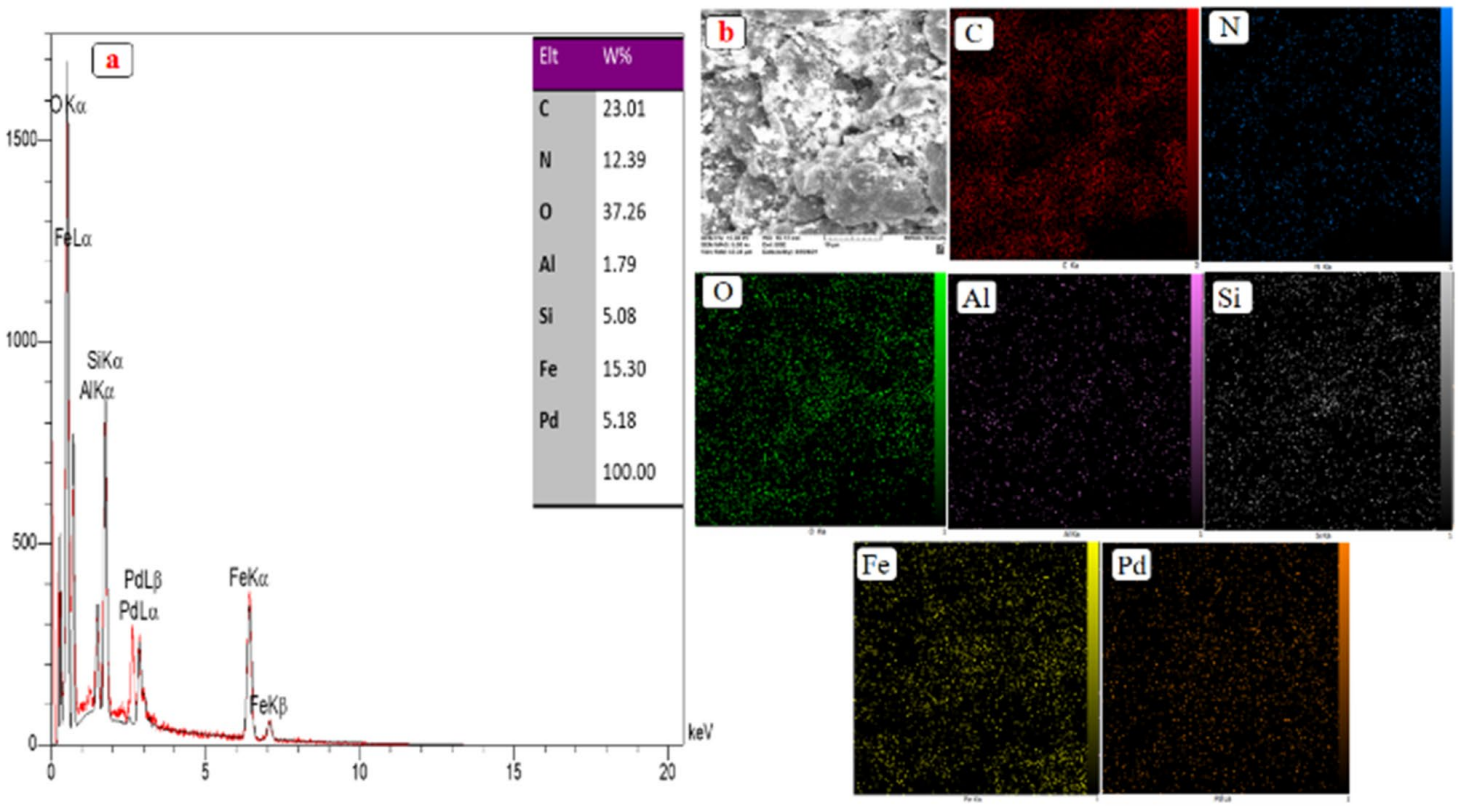
Corresponding EDX spectrum of Fe-Ben/PVP-DVB/Pd nanocatalyst (**a**), elemental mapping patterns of C, N, O, Al, Si, Fe, and Pd atoms (**b**).

Figure 6 shows an SEM image of the prepared nanocatalyst. As can be seen, Pd and Fe_3_O_4_ nanoparticles have been successfully dispersed and deposited on the surface of the nanocatalyst and one of the factors of this dispersion can be considered the effective presence of PVP-DVB. To further confirm the presence of nanoparticles and also to prove the presence of other elements in the structure of the nanocatalyst, the EDX technique (Fig. 7a) and mapping (Fig. 7b) were used. The results indicated the presence of Fe, Al, C, N, Si, Pd, and O elements in the nanocatalyst structure. Also, the amount of loaded palladium in the Fe-Ben/PVP-DVB magnetic composite was determined using ICP spectroscopy and was about 1.588%. This difference shows that most of the Pd NPs are dispersed on the surface of the nanocatalyst. On the other hand, in order to determine the stability of the nanocatalyst, the amount of Pd loaded after recycling was also measured. ICP-OES analysis showed that the recycled nanocatalyst contained 1.092% Pd and this good stability led to its high activity.

### VSM

**Figure 8:**
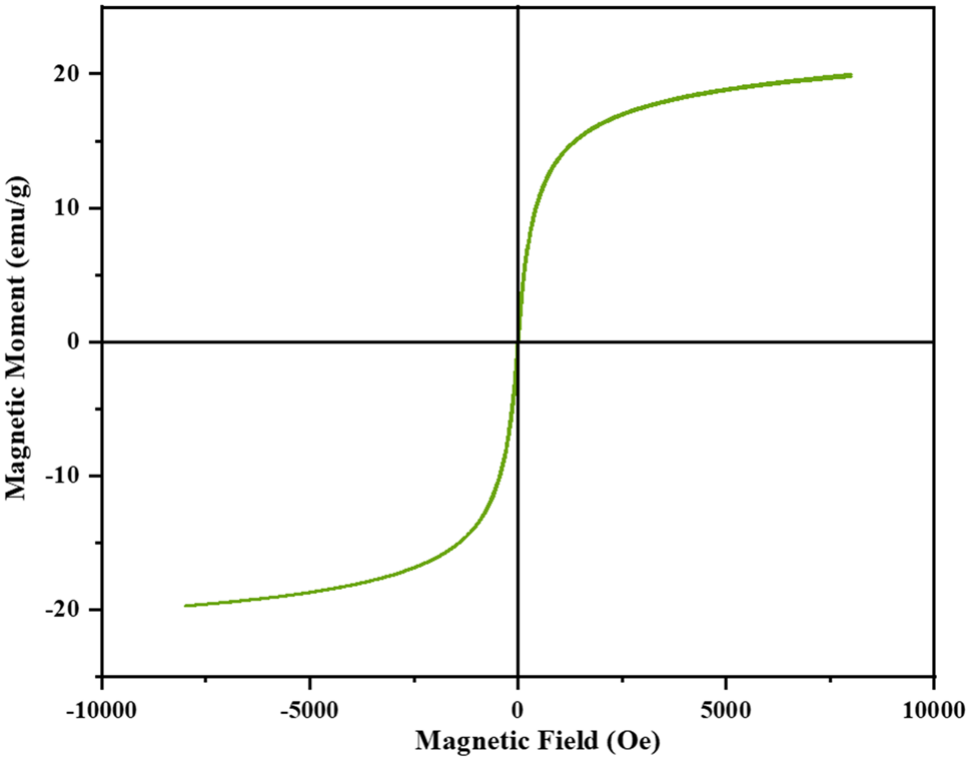
Magnetization curve of Fe-Ben/PVP-DVB/Pd nanocatalyst.

To investigate the magnetic properties of the prepared nanocatalyst, the sample magnetism was measured and examined with a vibrating sample magnetometer (VSM) from − 8000 Oe to + 8000 Oe at room temperature. Figure 8 carefully shows that the magnetic induction (Hc) and the magnetic residual (Mr) properties are zero. Therefore, nanocatalyst has superparamagnetic properties^23^. On the other hand, the amount of magnetic saturation (M_S_) for bare Fe_3_O_4_ nanocrystals is about 70 emu g^−1^ ^24^, which has been reduced to 20 emu g ^-1^ for Fe-Ben/PVP-DVB/Pd. In other words, the presence of polymer and bentonite in the nanocatalyst structure has reduced its magnetic saturation in compared to bare Fe_3_O_4_^15^. But the same large magnetic value demonstrates that this nanocatalyst still owns a good magnetic permeability and can be easily removed and reused several times by an external magnet without a significant reduction in its magnetic property.

### XRD pattern analysis

**Figure 9:**
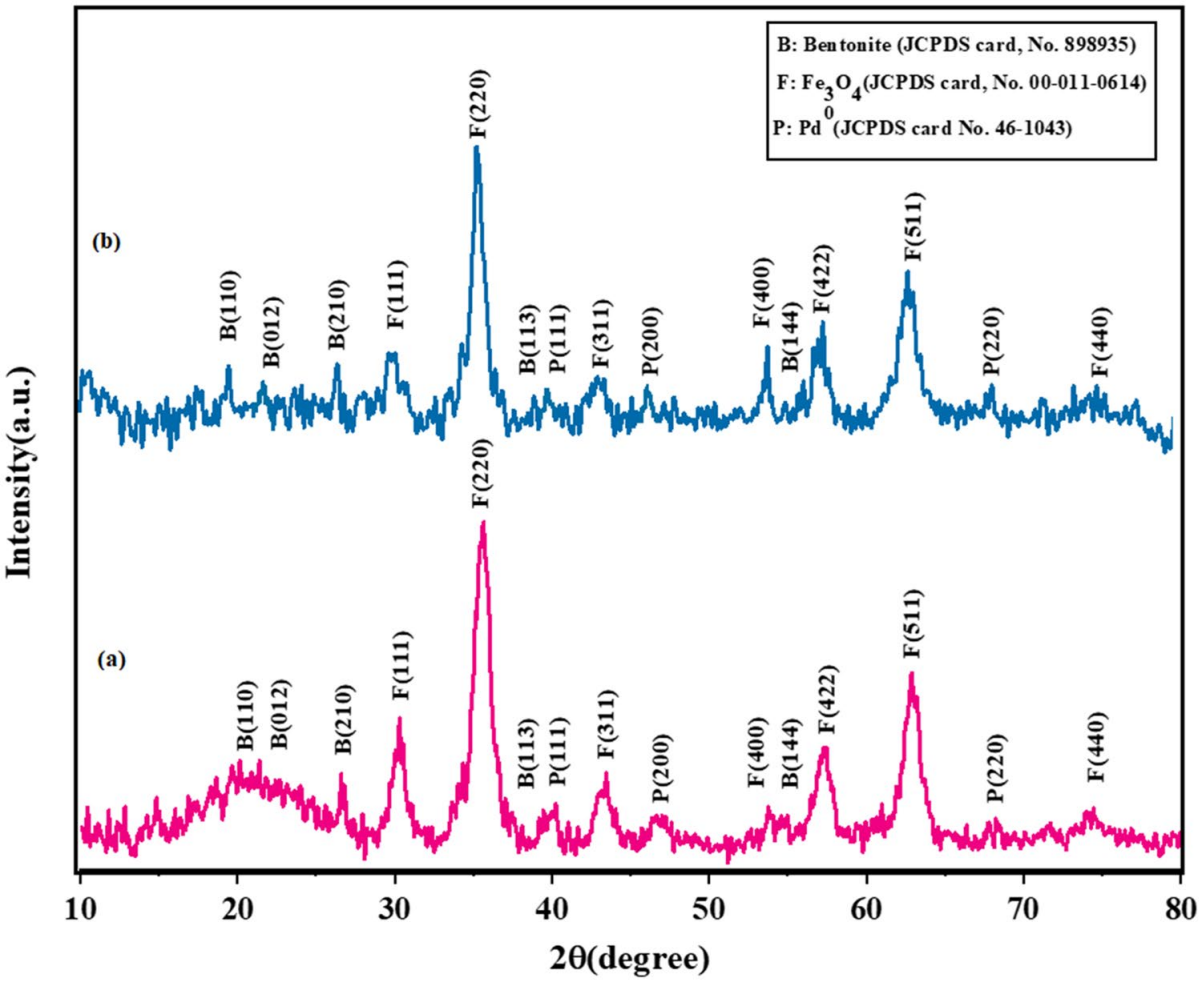
XRD pattern of (**a**) fresh, and (**b**) reused Fe-Ben/PVP-DVB/Pd nanocatalyst.

The crystal phase and purity of the prepared nanocatalyst were analyzed using XRD. This pattern for bentonite as well as Fe_3_O_4_ and Pd (0) nanoparticles stabilized on the Fe-Ben/PVP-DVB/Pd nanocatalyst is shown in Fig. 9a. The peaks shown in the diffraction angle 2θ = 20.2°, 22.5°, 26.6°, 39.4°, and 54.7° correspond respectively with plates (110), (012), (210), (113), and (144) of bentonite (JCPDS card, No. 898935)^25,26^. Also, strong diffraction peaks are observed at 2θ = 30.3°, 35.6°, 43.4°,53.8°, 57.2°, 63.05°, and 74.04°, which correspond to crystalline plates with miller indexes of (111), (220), (311), (400), (422), (511) and (440) respectively^27^. These peaks conform to the standard Fe_3_O_4_ crystal magnetite pattern and are attributed to the crystal plates of its face-centered cubic spinel structures (JCPDS card, No. 00-011-0614). On the other hand, the three specified peaks at 40.1°, 46.7°, and 68.2° correspond to (111), (200), and (220) plates of Pd (0) nanoparticle (JCPDS card No. 46-1043)^28^. These peaks clearly confirm the conversion of Pd (II) to Pd (0) and show that Pd NPs have been successfully stabilized on the composite surface. Also, Fig. 9b shows the crystallographic structure of Fe-Ben/PVP-DVB/Pd nanocatalyst after recycling, which was stable and managed to maintain its structure.

### X-ray photoelectron spectroscopy (XPS)

**Figure 10:**
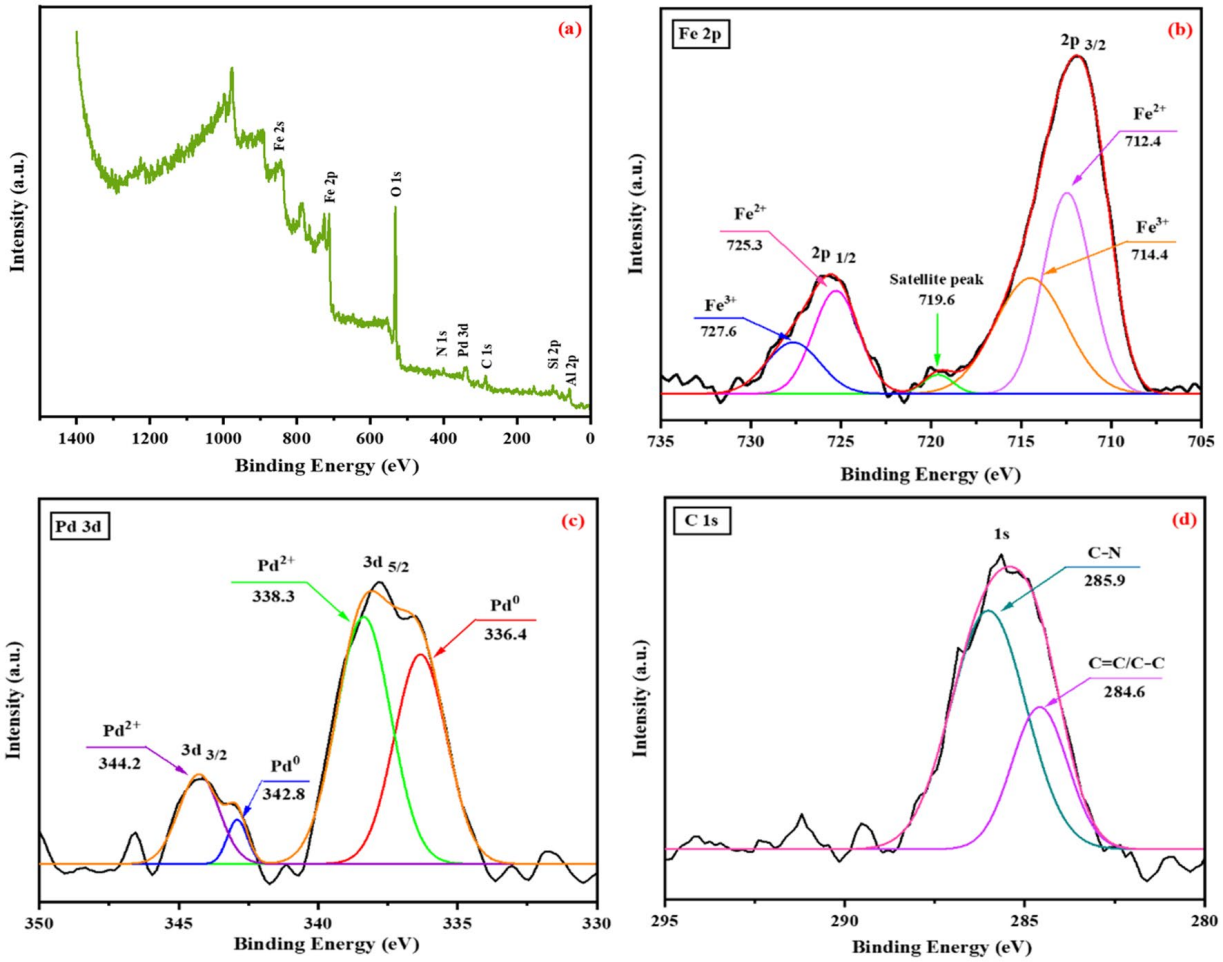
(**a**) XPS spectrum of Fe-Ben/PVP-DVB/Pd nanocatalyst. (**b**–**d**) High-resolution XPS spectra of Fe 2p, Pd 3d, and C1s.

In order to investigate the surface composition and capacity of each element, an X-ray photoelectron spectrum (XPS) was obtained from Fe-Ben/PVP-DVB/Pd nanocatalyst (Fig. 10). The peaks related to Si 2p, Al 2p, Fe 2p, Pd 3d, N 1s, C 1s, and O 1s are present in the investigated XPS spectrum (Fig. 10a). The high-resolution Fe 2p spectrum in Fig. 10b shows five distinct peaks. The peaks at 712.4 and 725.3 eV correspond to Fe^2+^, while those at 714.4 and 727.6 eV are assigned to Fe^3+^. The weak peak observed at 719.6 eV was related to the satellite peak and confirmed the purity and successful formation of the Fe_3_O_4_ phase in the nanocatalyst^29^. Figure 10C shows the Pd 3d spectrum with high resolution. In this image, four separate peaks can be seen. The peaks at 338.3 and 344.2 eV are assigned to Pd^2+^ or PdO, while the peaks at 336.4 and 342.8 eV correspond to metallic Pd^0^^30^. On the other hand, in the spectrum of C1s (Fig. 10d), two peaks can be seen with high resolution. The peak at 284.6 eV corresponds to the binding energy of C=C/C–C and the peak at 285.9 eV is specific to the binding energy of C-N, which confirms the successful presence of PVP-DVB in the nanocatalyst^31^.

### Thermogravimetric analysis (TGA)

**Figure11:**
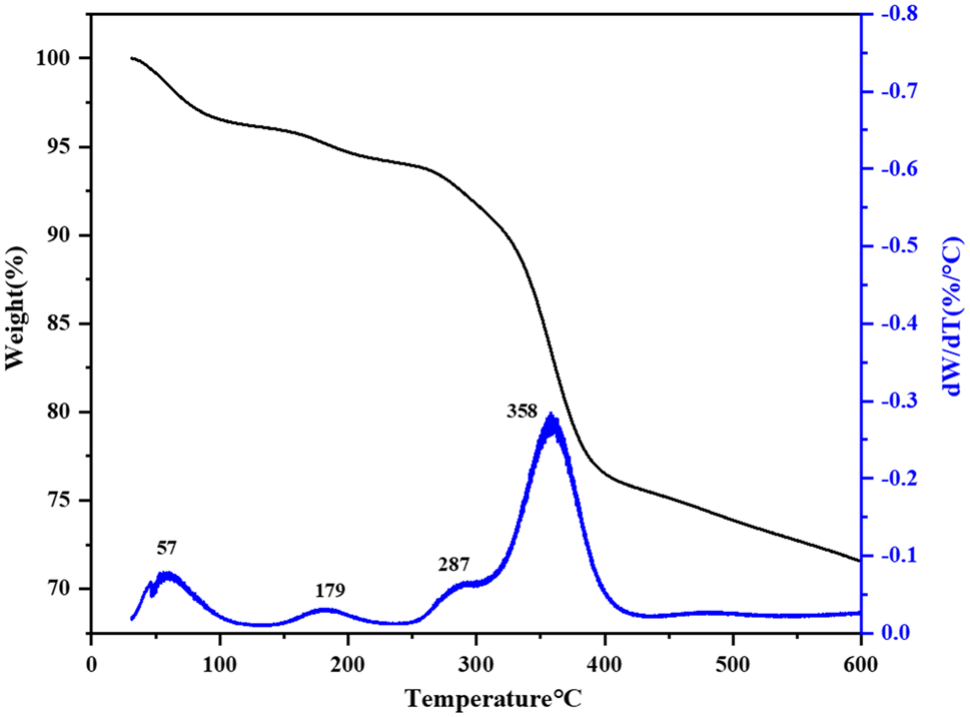
TGA and DTG thermograms of Fe-Ben/PVP-DVB/Pd nanocatalyst at a heating rate of 10 °C/min.

The TGA and dTGA diagrams for the prepared nanocatalyst were recorded by heating the sample at a rate of 10 °C per minute (Fig. 11). The endothermic peak at 57 °C and 179 °C show weight loss due to the removal of physical moisture on the surface of the nanocatalyst and interstructural water respectively^32^. The maximum weight loss at 358° C is related to the degradation of the main chain of PVP-DVB and the peak at 287 °C is related to the degradation of other organic compounds in the nanocatalyst structure^20^. This analysis shows that 28.42% of weight loss can be attributed to the removal of the polymer. This composite has a high thermal resistance of up to 400 °C. Therefore, it can be said that Fe-Ben/PVP-DVB/Pd is a high-temperature nanocatalyst whose chemical structure is well preserved.

### Catalytic activity of Fe-Ben/PVP-DVB/Pd in Suzuki–Miyaura reactions

To consider the catalytic behavior of Fe-Ben/PVP-DVB/Pd, the cross-linking reaction of phenylboronic acid and iodobenzene was selected as a model reaction, and to achieve the desired conditions different parameters of this reaction were examined (Table 1). The reaction was first studied in the absence of the nanocatalyst and the results showed that the reaction progress was zero even after 10 h (Table 1, entry 1).

**Table 1:** Optimized Suzuki‐Miyaura coupling reaction conditions.


Entry	Base	Solvent	Pd (mol%)	Time (min)	Temp (°C)	Yield (%)^a^
1	K_2_CO_3_	EtOH/H_2_O(1:1)	0	180	80	0
2	K_2_CO_3_	EtOH/H_2_O(1:1)	0.3	30	80	98
3	K_2_CO_3_	EtOH/H_2_O(1:1)	0.3	15	80	98
4	K_2_CO_3_	EtOH/H_2_O(1:1)	0.3	90	40	65
5	K_2_CO_3_	EtOH/H_2_O(1:1)	0.3	90	r.t	50
6	K_2_CO_3_	EtOH/H_2_O(1:1)	0.3	15	60	98
7	K_2_CO_3_	H_2_O	0.3	30	60	50
8	K_2_CO_3_	EtOH	0.3	30	60	50
9	K_2_CO_3_	MeCN	0.3	30	60	60
10	K_2_CO_3_	THF	0.3	30	60	45
11	K_2_CO_3_	DMSO	0.3	30	60	68
12	K_2_CO_3_	DMF	0.3	30	60	70
13	K_2_CO_3_	EtOH/H_2_O(1:1)	0.2	15	60	77
14	K_2_CO_3_	EtOH/H_2_O(1:1)	0.15	15	60	56
15	No base	EtOH/H_2_O(1:1)	0.3	30	60	4

Reaction condition: phenylboronic acid (0.6 mmol), base (0.4 mmol), Iodobenzene (0.5 mmol), solvent (2 mL), nanocatalyst (0.3 mol%).

^a^Isolated yield.

The reaction temperature is very important in catalytic processes therefore, different temperatures were investigated and the best performance was obtained at 60 °C (Table 1, entry 2–6). In the next step, the solvent with highly effective and at the same time more compatible with the principles of green chemistry was selected. A mixture of H_2_O/EtOH (1:1) was selected as an effective solvent with high performance (Table 1, entry 6–12). On the other hand, different amounts of nanocatalyst were used for optimization and 0.3 mol% of it showed high efficiency (Table 1, entry 3 and 13–14). In the last step, the reaction conditions of the model without the presence of a base were investigated and the results showed that the base has an essential role (K_2_CO_3_) in the Suzuki reactions (Table 1, entry 15). In the end, the optimal reaction conditions were determined as follows: 0.3 mol% Fe-Ben/PVP-DVB/Pd nanocatalyst, 0.073 g phenylboronic acid (0.6 mmol), 0.06 g potassium carbonate (2 mmol), 0.5 mmol aryl halide (0.5 equivalents) and finally 2 mL of water/ethanol solvent mixture (v/v = 1:1) at 60 °C for 15 min. The generality of this method in the reaction of phenylboronic acid with various aryl halides was investigated, the results are shown in Table 2 and as expected, aryl iodides and aryl bromides are more reactive than aryl chlorides. Also, both electron-donating and electron-withdrawing groups on aryl bromides showed good yields, turnover frequency (TOF), and turnover number (TON) in the presence of Fe-Ben/PVP-DVB /Pd nanocatalyst.

**Table 2:** Synthesis of biphenyl with different aryl halides under optimal conditions in the presence of Fe-Ben/PVP-DVB/Pd nanocatalyst.


Entry	Reactant	Time (h)	Product	Yield (%)^a^	TON	TOF
1		0.25		98	326.6	1306.4
2		0.25		93	310	1240
3		0.25		96	320	1280
4		0.25		98	326.6	1306.4
5		0.25		90	300	1200
6		0.25		65	216.6	886.4
7		0.25		78	260	1040
8		1.5		87	290	193.3
9		2.5		75	250	100
10		2.5		70	233.3	93.32

Reaction condition: 0.6 mmol phenylboronic acid, 0.4 mmol K_2_CO_3_, 0.5 mmol of aryl halide in 2 mL solvent (EtOH/H_2_O1:1), and in the presence of 0.3 mol% Pd nanocatalyst at 60 °C.

^a^Isolated yield. TON: (yield of product/per mol of Pd). TOF: (TON/time of reaction).

In Table 3, the catalytic role of Fe-Ben/PVP-DVB/Pd nanocatalyst in the present study was compared with a number of nanocatalysts reported in previous studies. According to the table, it can be seen that Fe-Ben/PVP-DVB/Pd nanocatalyst in the model reaction of bromobenzene with phenylboronic acid has higher product yield, shorter reaction time, and milder reaction conditions than other catalysts.

**Table 3:** Comparison of the performance of Fe-Ben/PVP-DVB/Pd nanocatalyst in the model Suzuki–Miyaura coupling reaction to some Pd catalysts reported in the literature.

Entry	Catalyst	Condition	Yield (%)	References
	GO-NH_2_-Pd(II)	EtOH/H2O,4 h, 60 °C	73	^33^
1	Pd/SiO_2_	H2O/DMF, 3 h, 200 °C	65	^34^
2	Pd@Fe_3_O_4_@C	DMF /EtOH,4 h, 100 °C	99	^35^
3	G–Ni/Pd	H2O/DMF,30 min,110 °C	78	^36^
4	m-f-MWCNTs@chitosan NHC-Pd	H2O/EtOH,3 h, 50 °C	94	^37^
5	Fe-Ben/PVP-DVB/Pd	H2O/EtOH,15 min, 60 °C	93	This work

**Figure 12:**
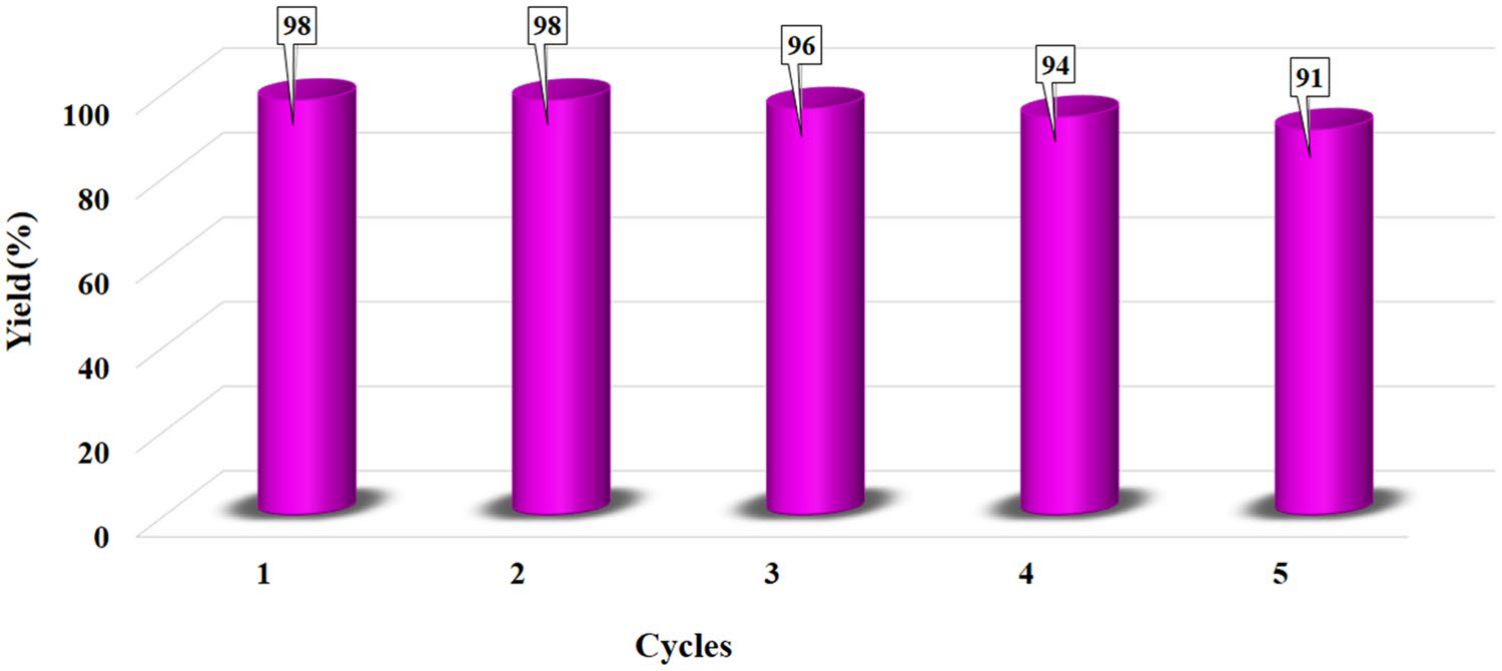
Magnetic separation and recycling of Fe-Ben/PVP-DVB/Pd nanocatalyst in the model reaction.

To evaluate the stability of the nanocatalyst, after the completion of the Suzuki reaction, the nanocatalyst was first removed from the reaction medium by a magnet, then rinsed several times with ethanol and water, and reused. This study showed that the nanocatalyst can be used for up to 5 cycles and its efficiency can be maintained without a significant decrease in performance (Fig. 12).

Also, the heterogeneous nature of Fe-Ben/PVP-DVB/Pd nanocatalyst was confirmed through a hot filtration test^38^. In the first step, a possible Suzuki coupling reaction of iodobenzene with phenylboronic acid was carried out in optimal conditions and after 7 min. The desired product was obtained with an efficiency of 72%. In the second step, the magnetic nanocatalyst was completely separated from the reaction medium by an external magnet, and the reaction continued for another 7 min without it. Investigations did not confirm any increase in product yield, indicating the heterogeneous nature of the Fe-Ben/PVP-DVB/Pd nanocatalyst.

### Catalytic activity of Fe-Ben/PVP-DVB/Pd in the reduction of 4-NP in water

**Figure 13:**
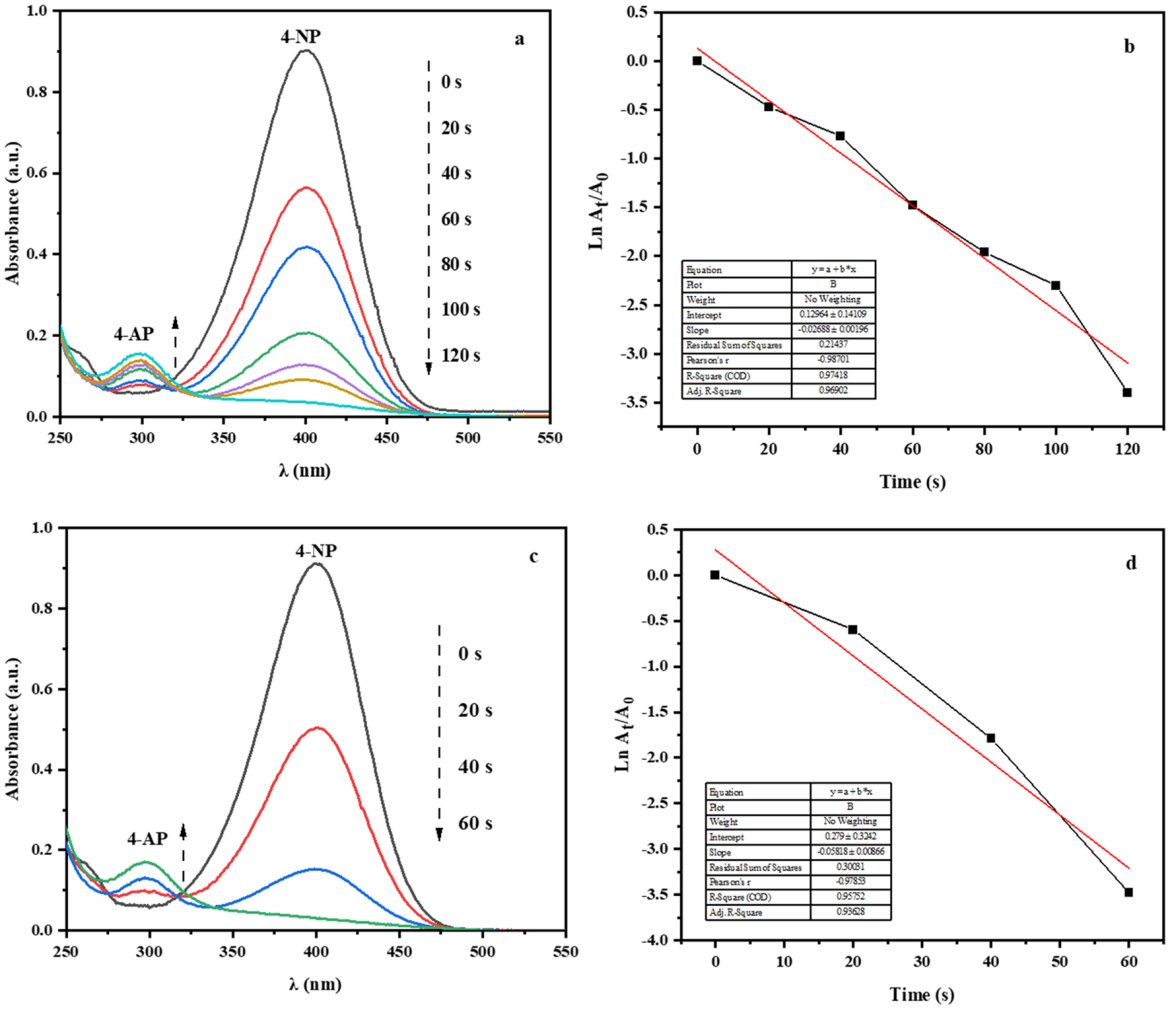
(**a**) Reduction of 4-NP in aqueous solution recorded every 20 s using Fe-Ben/PVP-DVB/Pd nanocatalyst (1 mg); (**b**) ln (A_t_/A_0_) versus reaction time for reduction of 4-NP. (**c**) Reduction of 4-NP in aqueous solution recorded every 20 s using Fe-Ben/PVP-DVB/Pd nanocatalyst (2 mg); (**d**) ln (A_t_/A_0_) versus reaction time for reduction of 4-NP.

The UV–vis spectroscopy was used to evaluate the performance of the nanocatalyst in the reducing reaction of 4-NP to 4-AP. This study was performed first with 2 mg and then 1 mg of Fe-Ben/PVP-DVB/Pd nanocatalyst in the presence of NaBH_4_ as a reducing agent and in the aqueous medium. As shown in Fig. 13a and c, at zero moments, the absorption peak of 4-NP appeared in the range of 400 nm. This peak disappears in 60 s for 2 mg of nanocatalyst and in 120 s for 1 mg of nanocatalyst. On the other hand, during the reaction process and over time, the peak of 4-AP also appears in the range of 300 nm. The reduction of the 4-NP compound to 4-AP can be seen by changing the color of the solution visually. The freshly prepared solution of 4-NP in the presence of the reducing agent NaBH_4_ has a light-yellow color, which gradually decreases and at the end of the reaction becomes completely colorless. Also, the linear relationships of ln (*A*_t_/*A*_0_) and reaction time (t) to reduce the p-NP compound are shown in Fig. 13b and d. This relationship is the pseudo-first-order and accordingly, the reaction rate constant was obtained at 0.0582 s^−1^ for 2 mg of nanocatalyst and 0.0269 s^−1^for 1 mg nanocatalyst.

## Conclusions

In this study, a new nanocatalyst based on bentonite clay as an effective support was synthesized and used at the reduction of 4-NP and Suzuki cross-coupling. The presented method is by the principles of green chemistry, successful, and with high efficiency. The presence of PVP cross-linked on the nanocatalyst substrate seems to plays an important and effective role in the stabilization and activity of Pd nanoparticles. The prepared Pd magnetic nanocomposite has a high stability at high temperatures and very little solubility in most organic solvents and so, it can be easily separated from the reaction medium by an external magnet after the end of the reaction and has reusability with slight deactivation after five cycles of reaction. Other advantages of this catalytic system include its mild reaction conditions, simple preparation method, and its remarkable response to aryl chlorides in the coupling reaction, which ultimately does not produce environmentally hazardous waste.

## Data Availability

All data generated or analyzed during this study are included in this published article.
